# The incidence of discordant clinical and genomic risk in patients with invasive lobular or ductal carcinoma of the breast: a National Cancer Database Study

**DOI:** 10.1038/s41523-021-00366-x

**Published:** 2021-12-21

**Authors:** Mary Kathryn Abel, Amy M. Shui, Michelle Melisko, A. Jo Chien, Emi J. Yoshida, Elizabeth M. Lancaster, Laura Van ‘T Veer, Laura J. Esserman, Rita A. Mukhtar

**Affiliations:** 1 University of California, San Francisco School of Medicine, 533 Parnassus Avenue, San Francisco, CA 94143 USA; 2 Department of Surgery, University of California, San Francisco, 400 Parnassus Avenue, San Francisco, CA 94143 USA; 3 Department of Epidemiology & Biostatistics, University of California, San Francisco, 550 16th Street, San Francisco, CA 94158 USA; 4 Division of Hematology and Oncology, University of California, San Francisco, 400 Parnassus Avenue, San Francisco, CA 94143 USA; 5 Department of Radiation Oncology, University of California, San Francisco, 1600 Divisadero Street, San Francisco, CA 94115 USA; 6 Helen Diller Comprehensive Cancer Center, University of California, San Francisco, 1600 Divisadero Street, San Francisco, CA 94115 USA

**Keywords:** Breast cancer, Cancer prevention

## Abstract

When molecular testing classifies breast tumors as low risk but clinical risk is high, the optimal management strategy is unknown. One group of patients who may be more likely to have such discordant risk are those with invasive lobular carcinoma of the breast. We sought to examine whether patients with invasive lobular carcinoma are more likely to have clinical high/genomic low-risk tumors compared to those with invasive ductal carcinoma, and to evaluate the impact on receipt of chemotherapy and overall survival. We conducted a cohort study using the National Cancer Database from 2010–2016. Patients with hormone receptor positive, HER2 negative, stage I-III breast cancer who underwent 70-gene signature testing were included. We evaluated the proportion of patients with discordant clinical and genomic risk by histology using Kaplan-Meier plots, log-rank tests, and Cox proportional hazards models with and without propensity score matching. A total of 7399 patients (1497 with invasive lobular carcinoma [20.2%]) were identified. Patients with invasive lobular carcinoma were significantly more likely to fall into a discordant risk category compared to those with invasive ductal carcinoma (46.8% versus 37.1%, *p* < 0.001), especially in the clinical high/genomic low risk subgroup (35.6% versus 19.2%, *p* < 0.001). In unadjusted analysis of the clinical high/genomic low-risk cohort who received chemotherapy, invasive ductal carcinoma patients had significantly improved overall survival compared to those with invasive lobular carcinoma (*p* = 0.02). These findings suggest that current tools for stratifying clinical and genomic risk could be improved for those with invasive lobular carcinoma to better tailor treatment selection.

## Introduction

Genomic testing has revolutionized the care of breast cancer patients by identifying individuals with molecularly low-risk tumors who can safely avoid chemotherapy, thereby personalizing breast cancer treatment^1–3^. However, available assays have been validated only in patients with limited extent of disease. For patients with more advanced clinical factors such as larger tumors, involved lymph nodes, younger age, and higher tumor grade, “clinical risk” can conflict with “genomic risk.” In such discordant situations, the optimal management strategy is unknown.

The MINDACT trial sought to address this question by studying the effect of chemotherapy on patients with early-stage breast cancer and discordant risk^4^. In this trial, patients with clinical high risk but genomic low-risk tumors were randomized to receive chemotherapy or not based on either clinical or genomic risk status. For patients with up to three positive nodes, distant metastasis-free survival at 5 years was not significantly different with or without chemotherapy. Subsequent evaluation with longer follow-up time has shown a potential small improvement in recurrence risk with chemotherapy treatment in the subset of women under the age of 50^5^. Consequently, the optimal management strategy for patients with discordant clinical and genomic risk remains incompletely understood, as subsets of patients with high clinical but low genomic risk may derive differential benefit from treatments such as cytotoxic chemotherapy or ovarian suppression.

One potential subset is individuals with invasive lobular carcinoma (ILC) of the breast. ILC affects 10–15% of all breast cancer patients and is a unique tumor type characterized by the lack of adhesion protein E-cadherin^6,7^. Although typically indolent and slow-growing, ILC often presents with higher stages of disease due to lower sensitivity of standard breast imaging tools^8–12^. Moreover, several investigators have found decreased responsiveness to chemotherapy in those with ILC histology^13,14^. Those with ILC also have high cumulative risk of recurrence, which may occur many years after initial diagnosis^15–17^. However, studies of genomic assays like the 70-gene signature (MammaPrint) and the 21-gene recurrence score (Oncotype Dx) show that a higher proportion of ILC patients have tumors characterized as low or intermediate risk compared to invasive ductal carcinoma (IDC)^18,19^.

Given the propensity for ILC to present at later stages and have molecular low risk by standard assays, we hypothesized that patients with ILC are more likely to fall into the discordant risk category of “clinical high/genomic low” compared to patients with hormone receptor (HR) positive, human epidermal growth factor-2 receptor (HER2) negative IDC. Therefore, we had three objectives in this study: (1) to establish whether patients with ILC are more likely to have discordant clinical and genomic risk compared to patients with IDC, (2) to determine whether rates of chemotherapy use differ by histologic status among those with discordant risk, and (3) to evaluate potential differences in overall survival (OS) by histology and treatment type in those with discordant risk.

## Results

### Study cohort

**Fig. 1:**
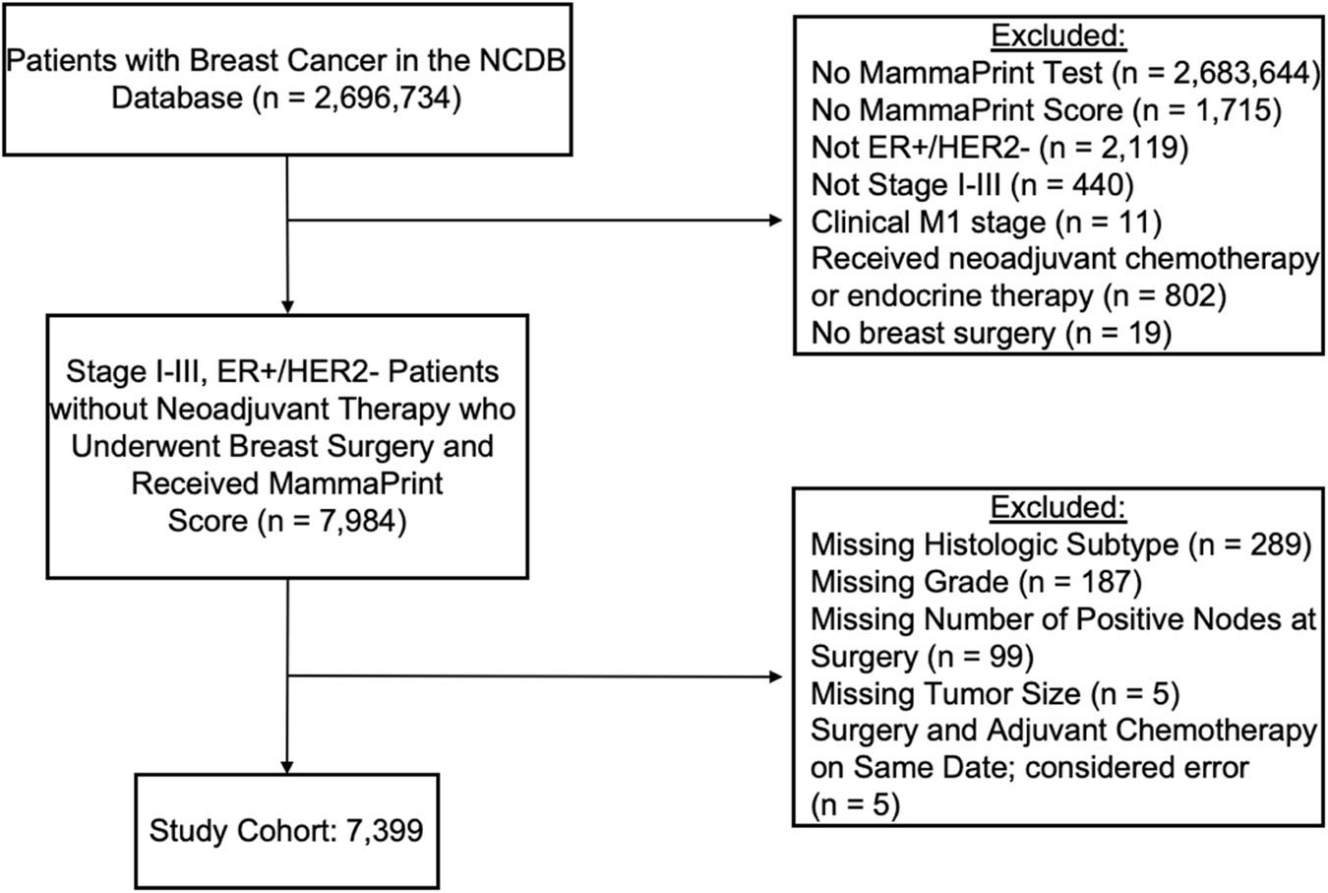
CONSORT diagram for study population. NCDB National Cancer Database, ER estrogen receptor, HER2 human epidermal growth factor receptor 2.

Overall, there were 738,762 HR-positive, HER2-negative patients included in the National Cancer Database (NCDB), of whom 9848 received 70-gene signature (MammaPrint) testing. There were no significant differences in the rate of 70-gene signature testing between patients with ILC compared to IDC. Of the 7399 patients meeting study criteria (Fig. 1), 5902 (79.8%) had IDC and 1,497 (20.2%) had ILC. Patients in the ILC cohort were slightly older than those in the IDC cohort (mean age 60.9 years versus 59.1, *p* < 0.001). Additionally, they were significantly more likely to present with higher stage disease and underwent mastectomy at higher rates (Table 1). The ILC tumors were significantly less likely to be grade 3, and chemotherapy was used less often in the ILC cohort compared to those with IDC. There were no differences in Charlson-Deyo score between the ILC and IDC cohorts.

**Table 1:** Clinicopathologic characteristics of study cohort.

	ILC (*N* = 1497)	IDC (*N* = 5902)	*P* Value
Age at diagnosis (years), mean (SD)	60.9 (10.4)	59.1 (11.1)	<0.001
Pathologic stage, *n* (%)			<0.001
I	727 (48.6%)	3744 (63.4%)	
II	664 (44.4%)	1993 (33.8%)	
III	106 (7.1%)	165 (2.8%)	
Tumor grade, *n* (%)			<0.001
1	345 (23.1%)	1534 (26.0%)	
2	1022 (68.3%)	3137 (53.2%)	
3	130 (8.7%)	1231 (20.9%)	
Clinical risk, *n* (%)			<0.001
Low	719 (48.0%)	3362 (57.0%)	
High	778 (52.0%)	2540 (43.0%)	
Genomic risk, *n* (%)			<0.001
Low	1085 (72.5%)	3435 (58.2%)	
High	412 (27.5%)	2467 (41.8%)	
Surgical therapy, *n* (%)			<0.001
Lumpectomy	803 (53.6%)	4018 (68.1%)	
Mastectomy	694 (46.4%)	1884 (31.9%)	
Adjuvant therapy, *n* (%)			
Chemotherapy	434 (29.5%)	2164 (37.4%)	<0.001
Endocrine therapy	1375 (93.4%)	5270 (91.0%)	0.003
Charlson-Deyo Score, *n* (%)			0.434
0	1287 (86.0%)	5052 (85.6%)	
1	177 (11.8%)	707 (12.0%)	
2	22 (1.5%)	114 (1.9%)	
≥3	11 (0.73%)	29 (0.49%)	

*ILC* invasive lobular carcinoma, *IDC* invasive ductal carcinoma.

### Discordant clinical and genomic risk

Patients with ILC were significantly more likely to fall into a discordant risk category compared to those with IDC, with nearly half of the patients with ILC having either clinical high/genomic low or clinical low/genomic high status (46.8% versus 37.1%, *p* < 0.001, Table 2). Among those with discordant risk, patients with ILC were far more likely to be clinical high/genomic low instead of clinical low/genomic high compared to those with IDC. In ILC patients with discordant risk, 76.1% had clinical high/genomic low status compared to 51.7% in IDC patients with discordant risk (*p* < 0.001). Additionally, among patients with ILC, those who were under 50 years old were significantly more likely to fall into the clinical high/genomic low-risk category compared to those aged ≥50 years (41.8% versus 34.4%, *p* = 0.026).

**Table 2:** Distribution of clinical and genomic risk categories by histology.

	ILC (*n* = 1497)	IDC (*n* = 5902)	*P* Value
Concordant risk, *n* (%)	797 (53.2%)	3715 (62.9%)	
Clinical low/Genomic low	552 (36.9%)	2305 (39.1%)	
Clinical high/Genomic high	245 (16.4%)	1410 (23.9%)	
Discordant risk, *n* (%)	700 (46.8%)	2187 (37.1%)	<0.001^1^
Clinical low/Genomic high	167 (11.2%)	1057 (17.9%)	
Clinical high/Genomic low	533 (35.6%)	1130 (19.2%)	<0.001^2^

Patients with ILC were significantly more likely to have discordance between clinical and genomic risk; among those with discordant risk, individuals with ILC were significantly more likely to have clinical high/genomic low-risk status.

^1^*P* value from chi-square tests for discordant risk vs. concordant risk.

^2^*P* value from chi-square tests for clinical high/genomic low status vs. clinical low/genomic high status.

*ILC* invasive lobular carcinoma, *IDC* invasive ductal carcinoma.

### Rates of chemotherapy use by risk category

The rates of chemotherapy use differed significantly between clinical and genomic risk categories. Those with genomic high risk (regardless of clinical risk) were significantly more likely to receive chemotherapy than those with genomic low risk (75.9% vs. 9.9%, *p* < 0.001). When grouped by clinical and genomic risk status, patients with ILC received chemotherapy at the same rate as patients with IDC except for those patients in the clinical high/genomic high-risk group (Table 3). Within this group, those with ILC were significantly less likely to receive chemotherapy compared to those with IDC (74.7% versus 80.9%, *p* = 0.0251). While these patients with ILC were slightly older than those with IDC (mean age 62 vs. 58 years respectively, *p* < 0.001), there was no difference in Charlson-Deyo Co-Morbidity Index between the two groups.

**Table 3:** Receipt of chemotherapy stratified by clinical and genomic risk category and histologic subtype.

Clinical/Genomic Risk Subgroup	Concordant risk				Discordant risk			
	Clinical low/Genomic low		Clinical high/Genomic high		Clinical low/Genomic high		Clinical high/Genomic low	
Histology	ILC *n* = 543	IDC *n* = 2247	ILC *n* = 241	IDC *n* = 1400	ILC *n* = 167	IDC *n* = 1036	ILC *n* = 522	IDC *n* = 1109
Chemotherapy *n* (%)	15 (2.8%)	70 (3.1%)	180 (74.7%)	1,133 (80.9%)	121 (72.5%)	726 (70.1%)	118 (22.6%)	235 (21.2%)
*P* value^1^	0.67		0.0251		0.54		0.52	

Among patients with clinical high/genomic high risk, individuals with ILC were significantly less likely to receive chemotherapy.

*ILC* invasive lobular carcinoma, *IDC* invasive ductal carcinoma.

^1^*P* values from two-sample test of proportions.

### Overall Survival

**Fig. 2:**
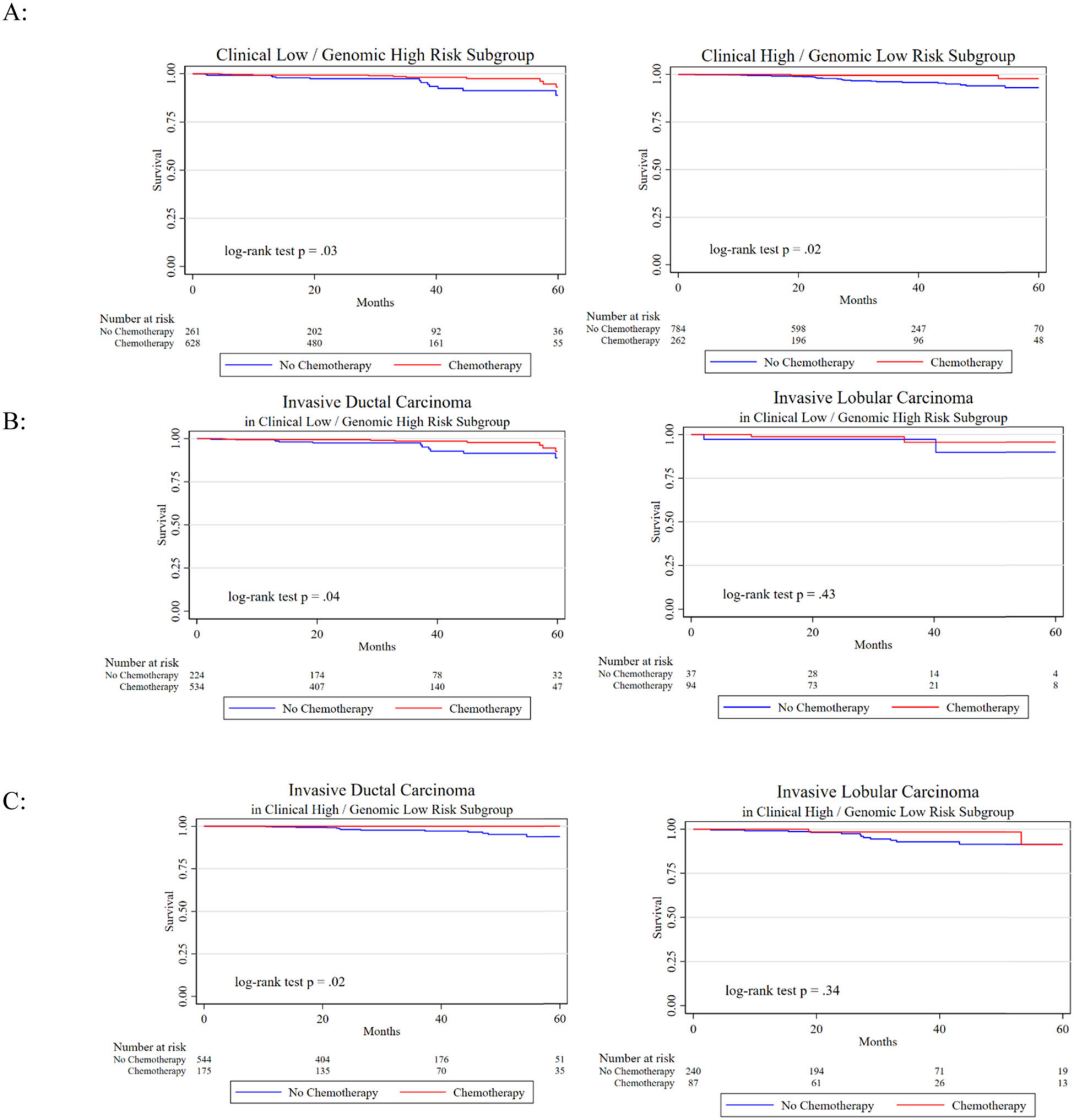
Survival plots by chemotherapy status, clinical/genomic risk subgroup, and histology. Survival plots by chemotherapy status in the clinical low/genomic high-risk subgroup and clinical high/genomic low-risk subgroup (**A**), in the clinical low/genomic high-risk subgroup by histology (**B**), and in the clinical high/genomic low-risk subgroups by histology (**C**).

The OS at 5 years in the study cohort was estimated to be 93.6%. In unadjusted analyses, receipt of chemotherapy was associated with significantly improved OS in both the clinical high/genomic low cases and the clinical low/genomic high cases (Fig. 2A, Supplementary Table 1). Within the clinical high/genomic low-risk group, receipt of chemotherapy was associated with a 79% lower mortality rate compared to those who did not receive chemotherapy (HR 0.21, 95% CI 0.05–0.90, *p* = 0.035). Similarly, within the clinical low/genomic high cohort, receipt of chemotherapy was associated with a 58% lower risk of death than those who did not receive chemotherapy (HR 0.42, 95% CI 0.19–0.94, *p* = 0.035).

Within the discordant risk subgroups, interaction analyses showed that the effect of chemotherapy on OS did not differ significantly by histology; however, from exploratory subgroup analyses, a significant improvement in OS was seen among patients with IDC who received chemotherapy, but not among those with ILC (Fig. 2B, C). In the clinical high/genomic low cohort, survival analyses were repeated in a propensity score-matched set to account for differences between those who did and did not receive chemotherapy. In this adjusted analysis that included 248 chemotherapy patients and 757 non-chemotherapy patients, receipt of chemotherapy was no longer associated with improved OS in any subgroup (Supplementary Table 1).

Survival outcomes were then evaluated in the cohort of patients who received chemotherapy to again account for unmeasured confounding. In the clinical high/genomic low cases who received chemotherapy, IDC histology was associated with improved OS compared to those with ILC (*p* = 0.02) (Supplementary Table 1, Supplementary Fig. 1). However, in the clinical low/genomic high cases who received chemotherapy, there was no difference in OS by histologic subtype.

## Discussion

In this NCDB study, we found that patients with HR-positive, HER2-negative ILC were significantly more likely to have clinical high/genomic low-risk status compared to those with HR-positive, HER2-negative IDC. This finding was particularly true for ILC patients under the age of 50 years, who had the highest rates of discordant risk status. The higher proportion of clinical high/genomic low-risk status among patients with ILC likely results from the tendency for patients with ILC to present at more advanced clinical stage in conjunction with high rates of “low-risk” status by the 70-gene signature^20^. Many prior studies have found that patients with ILC have larger tumors and more involved lymph nodes at diagnosis than patients with IDC^21–23^. Interestingly, in the IDC patients, those with discordant risk were equally likely to have clinical low/genomic high or clinical high/genomic low, highlighting that HR-positive, HER2-negative ILC and IDC have different characteristics.

This analysis is the first to evaluate this question and establish rates of discordant risk status by histologic subtype in a large, nationally representative database. While our ability to tailor treatment has increased for many patients with breast cancer, the optimal treatment strategy for those with discordant risk remains unclear. Recent randomized clinical trial data suggest that some subsets of clinical high/genomic low-risk patients may benefit from adjuvant chemotherapy. For example, the MINDACT study recently reported an 8-year update showing that younger patients with clinical high/genomic low risk derived significant, albeit small, improvement in distant metastasis-free survival with chemotherapy^5^. Additionally, the recently presented RxPonder data using the 21-gene recurrence score found that women with clinical high risk as defined by age under 50 and having 1–3 positive nodes but recurrence score of ≤25 had significantly improved invasive disease-free survival with chemotherapy^24^. Together, these studies suggest that among those with discordant clinical and genomic risk status, certain subsets derive more benefit from chemotherapy. As such, further investigation is needed to evaluate chemotherapy benefit by histologic status.

Among patients with clinical high/genomic high status, those with ILC were significantly less likely to receive chemotherapy than those with IDC. This finding is interesting, as prior data suggest that response to chemotherapy in patients with high genomic risk tumors does not differ by histology^16^. We suspect that this difference stems from a general belief that chemotherapy is less effective in ILC, which may contribute to provider reluctance and limit its use even in patients with genomic high-risk tumors. While patients with ILC in this group were significantly older than those with IDC (mean of 62 versus 58 years, *p* < 0.001), the clinical impact of this age difference is likely small and would not be expected to result in differential chemotherapy use, especially in the absence of difference in Charlson-Deyo Co-Morbidity Index. Importantly, in the clinical low/genomic high-risk tumors, the benefit from chemotherapy was equal in ILC and IDC. These findings support the need to develop lobular-specific risk stratification tools, since currently available tools appear to be interpreted differently by histologic status.

Finally, in our analyses evaluating the relationship between receipt of chemotherapy and OS, we found some evidence to support a potential differential benefit of chemotherapy by histologic subtype, although absolute differences were very small because OS in the entire cohort was 93.6% at 5 years. In the unadjusted analysis of clinical high/genomic low cases, only patients with IDC had significantly improved survival with chemotherapy, while patients with ILC did not. However, because patients in the NCDB were not randomized to receive chemotherapy, any benefit of chemotherapy could result from selection bias. To help account for this issue, we also evaluated OS by histology in a propensity score-matched cohort, and in an analysis restricted to those who received chemotherapy. In the propensity-matched cohort, we no longer found a difference in OS by histologic subtype. In the unadjusted analysis restricted to those who received chemotherapy (and were therefore all deemed fit enough to receive chemotherapy), we again found significantly improved survival in patients with IDC versus ILC. Although results are inconsistent, these findings suggest that current clinical trials evaluating optimal management strategies in patients with clinical high/genomic low-risk status should pursue histologic subtype-specific analyses, as results may not be applicable to all histology tumor types.

An important consideration in the interpretation of our findings is the use of OS as opposed to disease-free survival as the primary endpoint, as it is the only outcome available from the NCDB Participant User Files. Additionally, ILC and many HR-positive, HER2-negative tumors tend to recur beyond the first five years; as such, longer follow-up of these patients may yield different results with regards to the impact of chemotherapy on survival. It is possible that certain patients had differential access to molecular testing, leading to selection bias. For example, availability of molecular testing may have been limited in lower-resourced settings. We were lacking information regarding the type of chemotherapy or endocrine therapy administered, as well as duration of therapy. Finally, despite attempts to correct for the possibility of treatment selection bias, the retrospective nature of the NCDB limits the conclusions of these analyses. However, despite these limitations, our findings clearly show that patients with ILC are indeed significantly more likely to have discordant clinical and genomic risk. While the 70-gene signature has been shown to be independently prognostic in those with node-negative ILC, its predictive ability and the role of other prognosticators in node-positive ILC patients is unknown^18^. The recent identification of several ILC gene classifiers suggest the possibility that a prognostic or predictive tool specific to ILC could be developed. Another possible explanation for increased rates of discordance is that clinical staging might need tailoring for ILC, given its different growth pattern.

In conclusion, our study demonstrated that patients with ILC are significantly more likely to have discordant clinical and genomic risk compared to those with IDC when stratified by the 70-gene signature. Given the current active investigation into the best treatment strategy for patients with discordant risk profiles, histology-specific analyses of ongoing trials as well as further investigation into ILC specific prognostic and predictive tools are warranted.

## Methods

### Data source and study cohort

We utilized the NCDB, a national comprehensive clinical surveillance resource maintained by the American College of Surgeons and the American Cancer Society. The database represents over 70% of all newly diagnosed cancer cases in the United States and includes patient demographics, clinical information, and treatment outcomes^25,26^. For our analysis, Participants User Files from 2010–2016 were used. Due to the de-identified nature of the public-access user files, the study did not require institutional review board approval.

Because most ILC tumors are HR positive and HER2 negative, we limited our analysis to invasive tumors with this receptor subtype. We excluded patients with stage IV or unknown stage disease, those who received neoadjuvant therapy, individuals who did not undergo surgery for their breast cancer, and those who were missing critical clinical information including histologic subtype, molecular testing, HR status, tumor grade, number of positive lymph nodes on pathology, tumor size, and timing of chemotherapy or endocrine therapy.

### Clinical measures

Our primary outcome of interest was the rate of discordant clinical and genomic risk status by histologic subtype (ILC vs. IDC). Clinical risk was defined as high or low based on the criteria in the MINDACT trial, which used a modified version of the Adjuvant! Online calculator^4^. This clinical risk score assessment includes tumor size, grade, number of positive nodes at surgery, and HR and HER2 status. Genomic risk was assigned as high or low based on 70-gene signature scores as documented in the NCDB. Patients were considered to have “discordant” results if they had either clinical high/genomic low or clinical low/genomic high status. Patients had “concordant” results if they had either clinical high/genomic high or clinical low/genomic low status. Histology codes were used to identify cohorts, with the ILC cohort comprising those with codes for ILC or mixed ILC/IDC (histology codes 8520 and 8524 if behavior was invasive), and the IDC cohort comprising codes for IDC or invasive mammary carcinoma not otherwise specified (histology codes 8500, 8501, 8502, 8503, and 8523 if behavior was invasive). Charlson-Deyo Co-Morbidity Index was recorded as a measure of severity of co-morbid conditions. Age was subdivided into under 50 years and over 50 years to estimate pre- and post-menopausal status, respectively.

### Statistical analysis

Patient characteristics were described and differences between histologic subtype were evaluated using chi-square tests for categorical variables and t-tests for continuous variables. The frequency of patients in each of the four clinical and genomic risk categories was described by histology and by receipt of chemotherapy. Kaplan–Meier plots, log-rank tests, and Cox proportional hazards models were used to assess the association between 60-month OS and receipt of chemotherapy in pre-specified risk subgroups. Within each subgroup, interaction analyses were performed, and further post-hoc secondary exploratory subgroup analyses by histology and receipt of chemotherapy were performed.

Chi-square and *t*-tests were used to determine characteristics associated with receipt of chemotherapy within the clinical high/genomic low-risk group. Covariates significantly associated with receipt of chemotherapy were included in a propensity score model using logistic regression, and propensity score matching was implemented. In a sample of 1046 patients in this discordant group, patients who had chemotherapy (*n* = 262) were matched to patients who did not have chemotherapy (*n* = 784) using optimal full propensity score matching. A maximum of ten non-chemotherapy patients were matched with each chemotherapy patient. Weighted matched standardized differences and variance ratios for the propensity score model covariates were used to assess sample balance after matching.

Hypothesis tests were two-sided, and the significance threshold was set to 0.05. Statistical analyses were performed using Stata 16 and SAS version 9.4.

### Reporting summary

Further information on research design is available in the Nature Research Reporting Summary linked to this article.

## Supplementary information

Supplementary Documents

Reporting Summary

## Data Availability

The data that support the findings of this study are available from the National Cancer Database (NCDB), but restrictions apply to the availability of these data, which were used under license for the current study and so are not publicly available. Data are however available from the authors upon reasonable request and with permission of the NCDB.

## Code availability

Analysis code is available from the corresponding author upon reasonable request.

## References

[1] Van’t Veer LJ (2002). Gene expression profiling predicts clinical outcome of breast cancer. Nature.

[2] Paik S (2006). Gene expression and benefit of chemotherapy in women with node-negative, estrogen receptor-positive breast cancer. J. Clin. Oncol..

[3] van de Vijver MJ (2002). A gene-expression signature as a predictor of survival in breast cancer. N. Engl. J. Med..

[4] Cardoso F (2016). 70-Gene signature as an aid to treatment decisions in early-stage breast cancer. N. Engl. J. Med..

[5] Cardoso F. et al. MINDACT: long-term results of the large prospective trial testing the 70-gene signature MammaPrint as guidance for adjuvant chemotherapy in breast cancer patients. *J. Clin. Oncol.* **506** (2020). 10.1200/jco.2020.38.15_suppl.506

[6] Sledge GW, Chagpar A, Perou C (2016). Collective wisdom: lobular carcinoma of the breast. Am. Soc. Clin. Oncol. Educ. B..

[7] Mamtani A, King TA (2018). Lobular breast cancer: different disease, different algorithms?. Surg. Oncol. Clin. N. Am..

[8] Johnson K, Sarma D, Hwang ES (2015). Lobular breast cancer series: imaging. Breast Cancer Res..

[9] Chen Z (2017). Invasive lobular carcinoma of the breast: a special histological type compared with invasive ductal carcinoma. PLoS ONE.

[10] Adachi Y. et al. Comparison of clinical outcomes between luminal invasive ductal carcinoma and luminal invasive lobular carcinoma. *BMC Cancer* **16** (2016). 10.1186/s12885-016-2275-4 10.1186/s12885-016-2275-4 PMC4807554 27015895

[11] Adler OB, Engel A (1990). Invasive lobular carcinoma. Mammographic pattern. RoFo Fortschr. auf dem Geb. der Rontgenstrahlen und der Neuen Bildgeb Verfahr..

[12] Thomas M, Kelly ED, Abraham J, Kruse M (2019). Invasive lobular breast cancer: a review of pathogenesis, diagnosis, management, and future directions of early stage disease. Semin. Oncol..

[13] Marmor S (2017). Relative effectiveness of adjuvant chemotherapy for invasive lobular compared with invasive ductal carcinoma of the breast. Cancer.

[14] Tamirisa N (2019). The impact of chemotherapy sequence on survival in node-positive invasive lobular carcinoma. J. Surg. Oncol..

[15] Mann RM (2010). The impact of preoperative breast MRI on the re-excision rate in invasive lobular carcinoma of the breast. Breast Cancer Res. Treat..

[16] Lips EH (2012). Lobular histology and response to neoadjuvant chemotherapy in invasive breast cancer. Breast Cancer Res. Treat..

[17] Stein R. G. et al. The impact of breast cancer biological subtyping on tumor size assessment by ultrasound and mammography - a retrospective multicenter cohort study of 6543 primary breast cancer patients. *BMC Cancer*. **16** (2016). 10.1186/s12885-016-2426-7 10.1186/s12885-016-2426-7 PMC4943017 27411945

[18] Beumer IJ (2016). Prognostic value of MammaPrint® in invasive lobular breast cancer. Biomark. Insights.

[19] Soliman H (2020). MammaPrint guides treatment decisions in breast cancer: results of the IMPACt trial. BMC Cancer.

[20] Mook S (2010). Metastatic potential of T1 breast cancer can be predicted by the 70-gene MammaPrint signature. Ann. Surg. Oncol..

[21] Findlay-Shirras LJ, Lima I, Smith G, Clemons M, Arnaout A (2020). Population trends in lobular carcinoma of the breast: the Ontario experience. Ann. Surg. Oncol..

[22] Wang J (2014). Outcomes of sentinel lymph node dissection alone vs. axillary lymph node dissection in early stage invasive lobular carcinoma: a retrospective study of the Surveillance, Epidemiology and End Results (SEER) database. PLoS ONE.

[23] Singletary SE, Patel-Parekh L, Bland KI (2005). Treatment trends in early-stage invasive lobular carcinoma: a report from the National Cancer Data Base. Ann. Surg..

[24] Kalinsky K. et al. 21-Gene Assay to Inform Chemotherapy Benefit in Node-Positive Breast Cancer. *N. Engl. J. Med.* Published online December 1, 2021. 10.1056/NEJMoa2108873. 10.1056/NEJMoa2108873 PMC9096864 34914339

[25] Bilimoria KY, Bentrem DJ, Stewart AK, Winchester DP, Ko CY (2009). Comparison of Commission on Cancer-approved and -nonapproved hospitals in the United states: implications for studies that use the National Cancer Data Base. J. Clin. Oncol..

[26] Boffa DJ (2017). Using the national cancer database for outcomes research a review. JAMA Oncol..

