# Supplementary material for: The incidence of discordant clinical and genomic risk in patients with invasive lobular or ductal carcinoma of the breast: a National Cancer Database Study
Source: NPJ Breast Cancer. 2021 Dec 21;7:156. doi: 10.1038/s41523-021-00366-x (PMC8692497; doi:10.1038/s41523-021-00366-x)
Supplement: Supplementary file 1 — Supplementary Documents [file 41523_2021_366_MOESM1_ESM.pdf]

Supplementary Figure 1: Survival plots by histology in patients who received chemotherapy and were clinical high/genomic low (left) or clinical low/genomic high (right). In unadjusted analyses of patients who received chemotherapy, histologic subtype was associated with differences in overall survival only among those with clinical high/genomic low tumors. Among those with clinical high/genomic low tumors, patients with ductal histology had significantly improved survival compared to those with lobular histology.

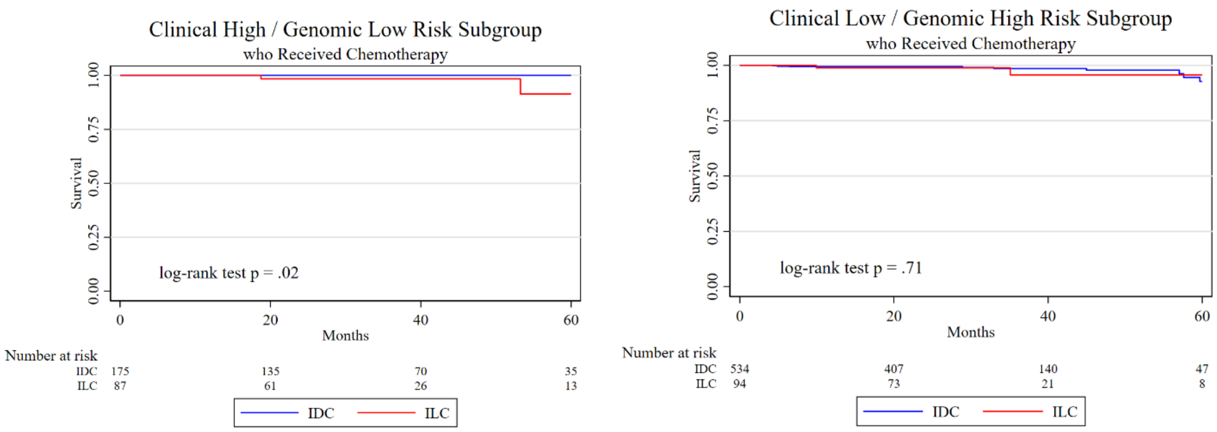

Supplementary Table 1: Summary of survival analyses by receipt of chemotherapy and by histologic subtype. In unadjusted analyses, receipt of chemotherapy was associated with significantly improved overall survival in both the clinical high/genomic low and the clinical low/genomic high subgroups. When stratified by histologic subtype, there was no chemotherapy benefit seen in patients with ILC in either risk subgroup. Among those who received chemotherapy and had clinical high/genomic low risk status, individuals with IDC had significantly improved survival compared to those with ILC, whereas no survival difference by histology was seen in the subgroup with clinical low/genomic high status.

Risk Group	Subgroup	Unmatched and Unadjusted Analysis	Propensity Score Matched Analysis	Unmatched and Unadjusted Analysis
		Chemotherapy vs. No Chemotherapy	Chemotherapy vs. No Chemotherapy	IDC vs. ILC
Clinical high/Genomic low	All	Log-rank p=0.02; HR 0.21, 95% CI 0.05-0.90, p=0.035	p=0.16	
	IDC	p=0.02	p=0.25	
	ILC	p=0.34	p=0.68	
	Chemotherapy			p=0.02[1]
Clinical low/Genomic high	All	Log-rank p=0.03; HR 0.42, 95% CI 0.19-0.94, p=0.035	N/A (groups are too different to match)	
	IDC	p=0.04		
	ILC	p=0.43		
	Chemotherapy			p=0.71

All p-values are from log-rank test except for those reported with a Cox model hazard ratio
ILC = invasive lobular carcinoma, IDC = invasive ductal carcinoma, HR = hazard ratio, CI = confidence interval

[1]Only 2 deaths total, both in ILC subgroup
